# Supplementary material for: Design and directed evolution of noncanonical β-stereoselective metalloglycosidases
Source: Nat Commun. 2022 Nov 11;13:6844. doi: 10.1038/s41467-022-34713-8 (PMC9652281; doi:10.1038/s41467-022-34713-8)
Supplement: Supplementary file 1 — Supplementary Information [file 41467_2022_34713_MOESM1_ESM.pdf]

Supplementary Information for

Design and Directed Evolution of Noncanonical β-Stereoselective Metalloglycosidases

by Jeong et al.

Table of contents

Supplementary Figures

Supplementary Figure 1. Geometric analysis of representative natural metallo-hydrolases.

Supplementary Figure 2. Geometric analysis of OmpF.

Supplementary Figure 3. The preparation of OmpF.

Supplementary Figure 4. The X-ray crystal structures of the OmpF variants.

Supplementary Figure 5. Steady-state esterase activities of the OmpF variants with pNPA.

Supplementary Figure 6. Steady-state β-lactamase activities of the OmpF variants with nitrocefin.

Supplementary Figure 7. Expression and translocation of the OmpF variants to the outer membrane.

Supplementary Figure 8. Steady-state glycosidase activities of the OmpF variants with 4-β-MUG.

Supplementary Figure 9. Screening of whole-cell mutant libraries.

Supplementary Figure 10. The screening results of (a) OmpF1, (b) OmpF1Y, and (c) OmpF2 as parent templates.

Supplementary Figure 11. Representative measurements of the stereoselective glycosidase activities of the OmpF variants.

Supplementary Figure 12. Measurements of the uncatalyzed hydrolytic rates of 4-β-MUG.

Supplementary Figure 13. The glycosidase activities of OmpF variants.

Supplementary Figure 14. The catalytic activities of OmpF variants with OG.

Supplementary Figure 15. The pH-dependent glycosidase activities of OmpF variants.

Supplementary Figure 16. Tandem LC/MS analysis of the trypsin-digested OmpF variants after incubation with CBE.

Supplementary Figure 17. Characterization of the glucosidase activity.

Supplementary Figure 18. Docking simulation of the OmpF variants with 4-β-MUG.

Supplementary Tables

Supplementary Table 1. Geometric analysis of the representative natural Zn-binding metalloproteins.

Supplementary Table 2. Custom-designed DNA primers for (a) site-directed mutagenesis and (b) saturation mutagenesis.

Supplementary Table 3. Crystallographic data and refinement statistics

Supplementary Table 4. The geometric parameters of the Zn-binding sites in the OmpF variants.

Supplementary Table 5. Steady-state kinetic analysis of the OmpF variants.

Supplementary Table 6. Glycosidase activities of the OmpF variants with 4-β-MUG.

Supplementary Table 7. ICP-MS results of OmpF2/E-R2.

Supplementary Table 8. A list of fragments conjugated with CBE in tandem LC/MS analysis.

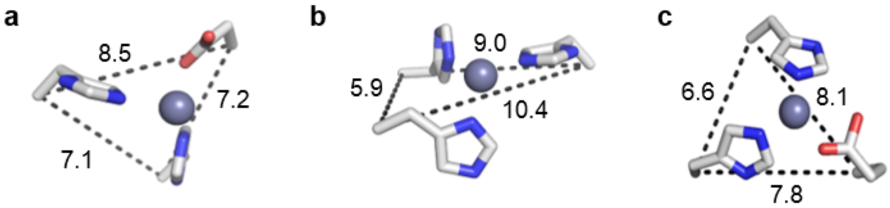

Supplementary Figure 1. Geometric analysis of representative natural metallo-hydrolases. (a) carbohydrate esterase (PDB 2CC0), (b) metallo-β-lactamase (PDB 2BC2), and (c) thermolysin (PDB 4TLN). The distance between the C_α atoms of the Zn-binding residues is shown in Å. Nitrogen and oxygen atoms are colored in blue and red, respectively. Zn ions are shown as spheres.

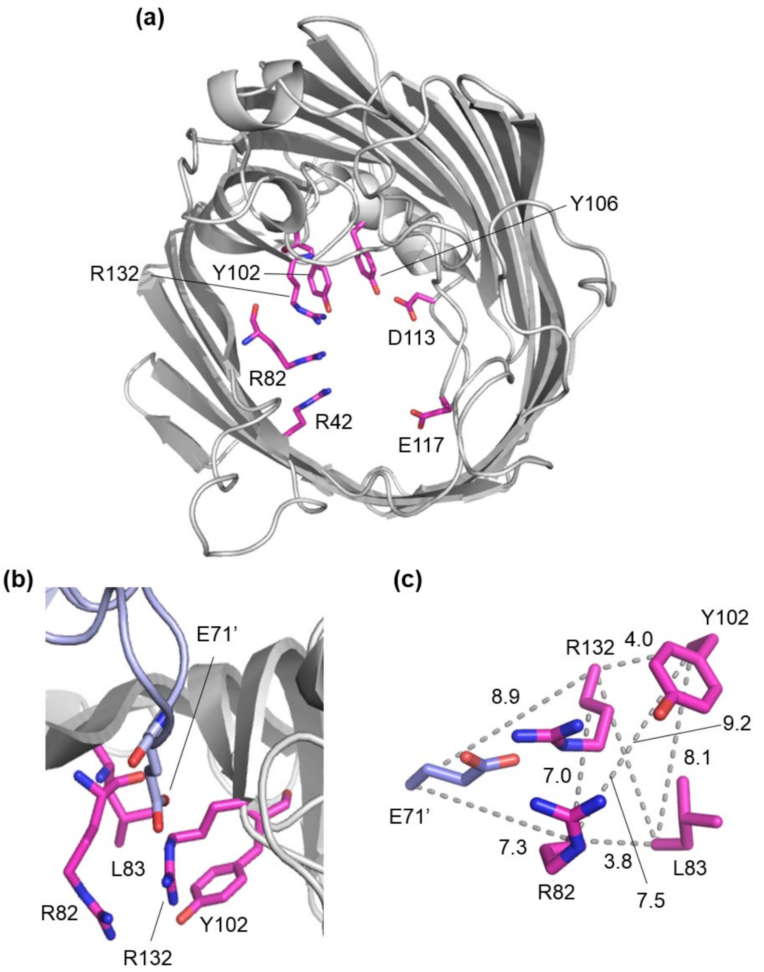

Supplementary Figure 2. Geometric analysis of OmpF. (a–b) Residues located in the constriction zone. Only the monomeric subunit is shown, for clarity. The adjacent protomer containing E71′ is colored in light purple. (c) Measurements of the C_α-C_α distance (Å) between the selected residues for the metal-binding sites.

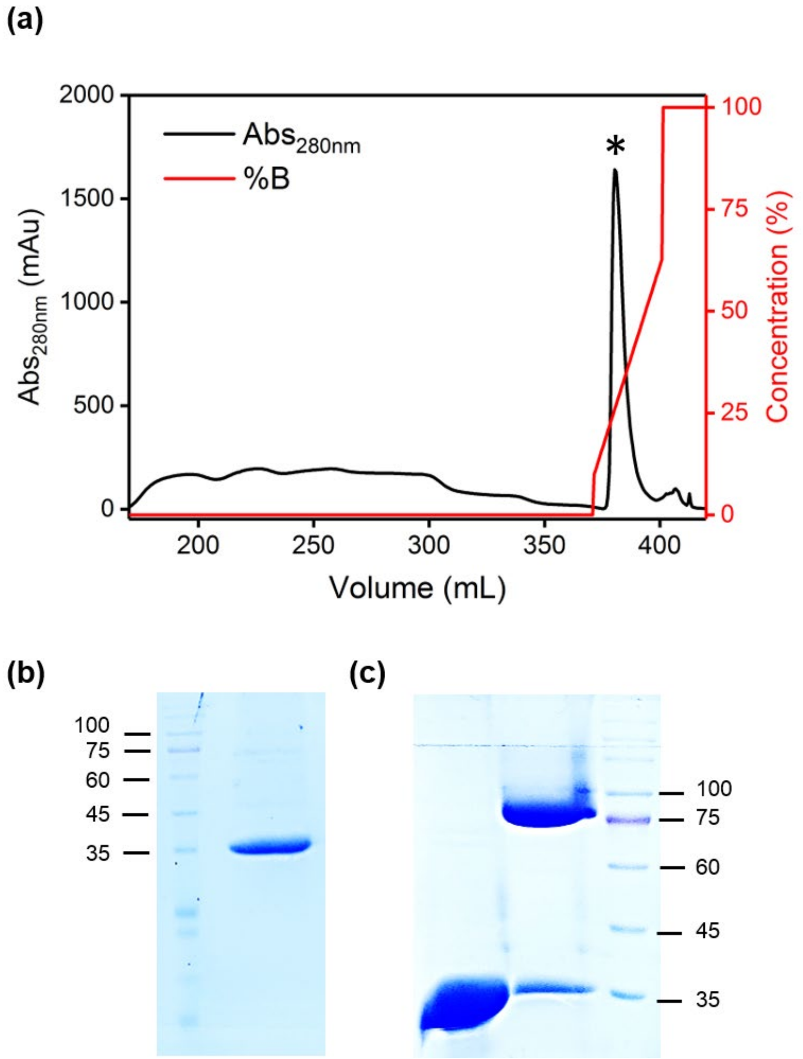

Supplementary Figure 3. The preparation of OmpF. (a) FPLC traces. An asterisk indicates the elution of the OmpF protein. (b) Representative SDS-PAGE of the purified unfolded OmpF protein. (c) OmpF refolding from an unfolded monomer (left) to a folded trimer (right). As reported previously in reference 18, a properly-refolded OmpF trimer runs faster (80 kDa) than its molecular size (110 kDa) in SDS-gel. The fraction of unfolded monomer was removed by trypsin digestion for further characterization. The data shown in (b) and (c) are representatives of at least ten trials, and all attempts to replicate were successful.

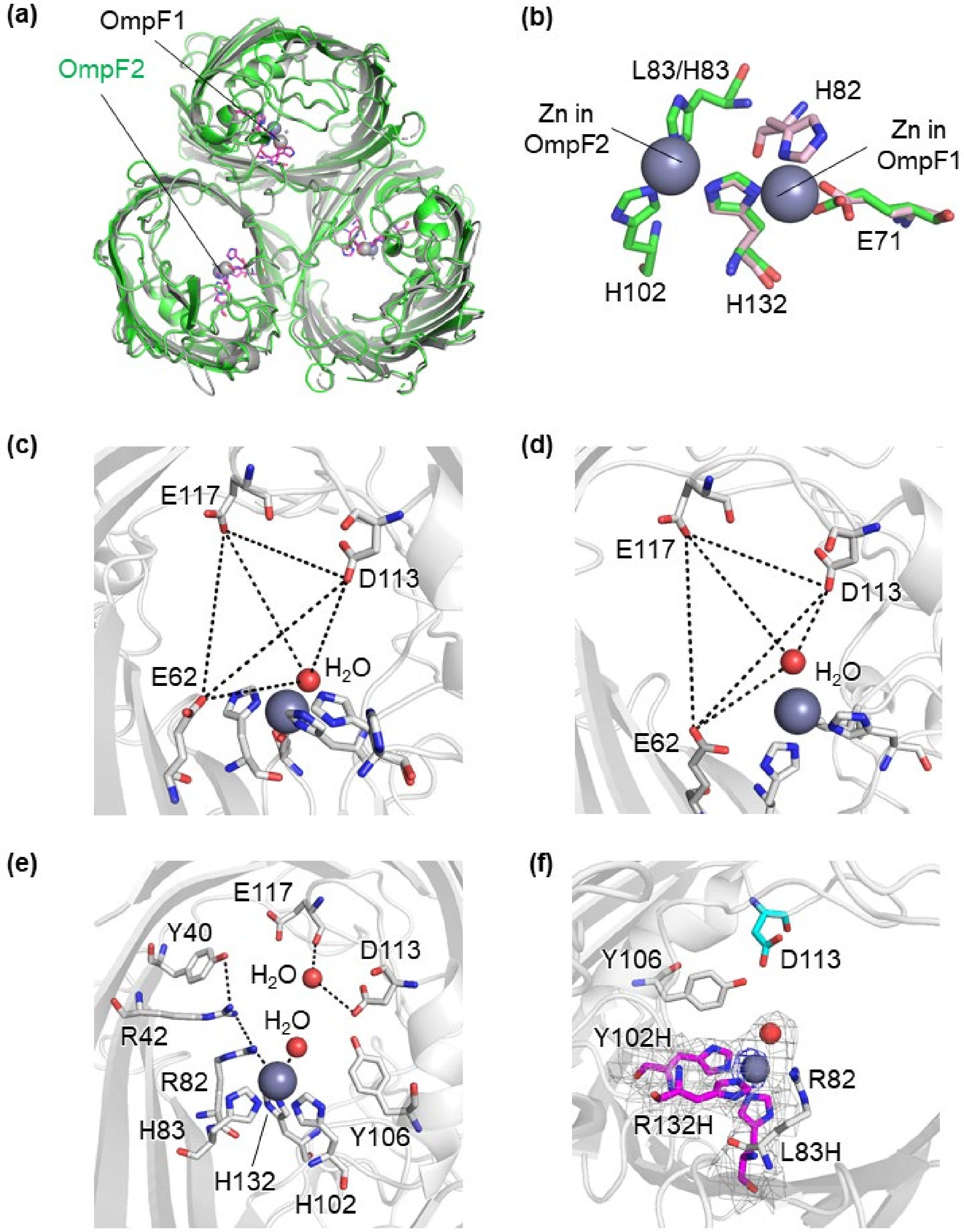

Supplementary Figure 4. The X-ray crystal structures of the OmpF variants. (a) The overlaid structures from the extracellular side. (b) The Zn-binding sites of the OmpF1 and OmpF2 variants, colored in light magenta and green, respectively. The acidic residues located near the Zn-binding site of (**c**) OmpF1 and (d) OmpF2 variants. The interatomic distances between key residues and motifs are summarized in Supplementary Table 4. (e) The hydrogen-bonding network observed in the Zn-binding site of OmpF2. Their distances are within 2.6–3.4 Å. (f) The overlaid electron density maps in OmpF2. The grey and blue grid represents the $2F_o - F_c$ electron density contoured at 1.0 σ and anomalous difference maps contoured at 3.0 σ, respectively.

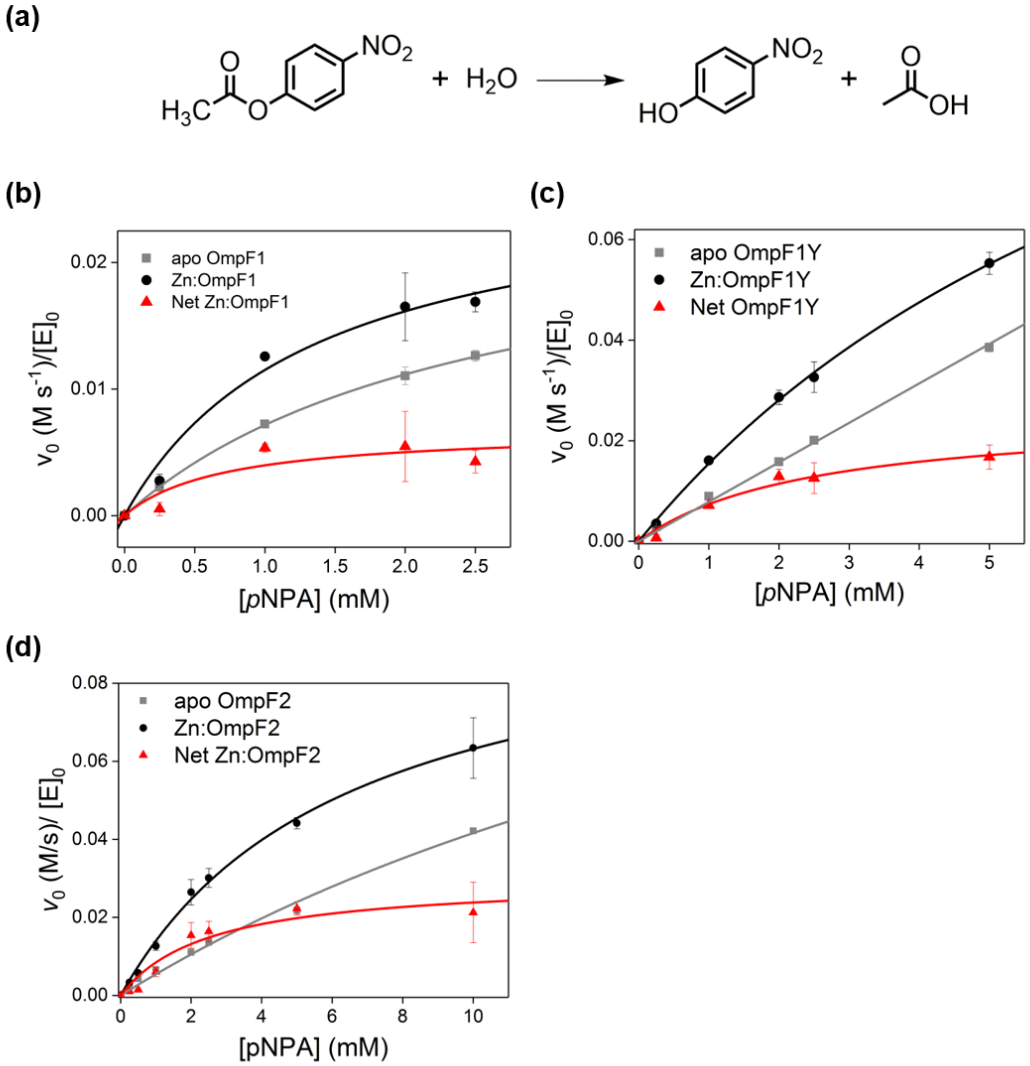

Supplementary Figure 5. Steady-state esterase activities of the OmpF variants with pNPA. (a) A reaction scheme. Michaelis-Menten kinetic analysis of (b) OmpF1, (c) OmpF1Y, and (d) OmpF2. The metal-dependent net activities were obtained by the subtraction of the observed activities measured using the apo-protein from those in the presence of Zn ions, in which free Zn ion alone exhibited no detectible activity. The data shown in (b), (c), and (d) represent the average and standard deviation of three independent experiments.

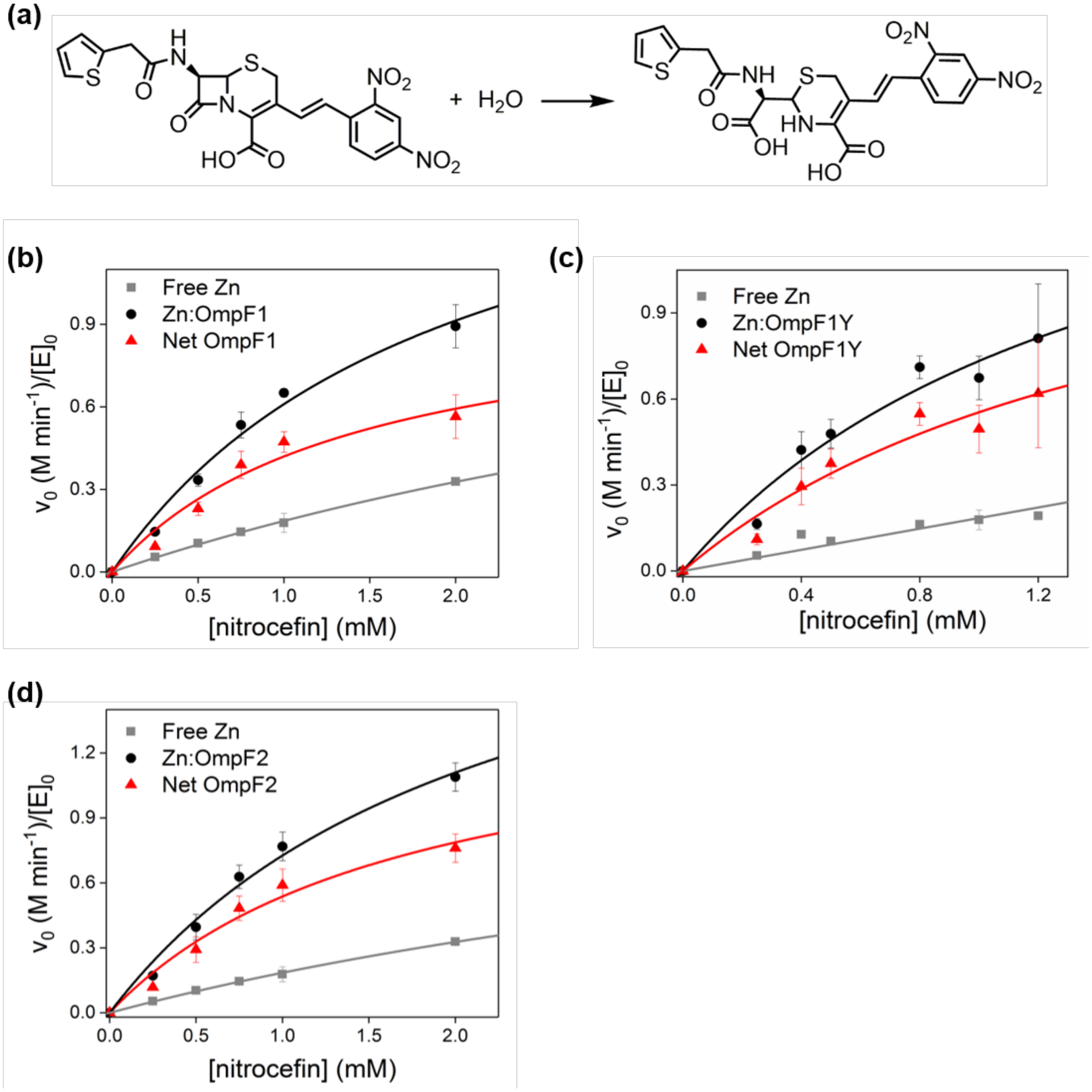

Supplementary Figure 6. Steady-state β-lactamase activities of the OmpF variants with nitrocefin. (a) A reaction scheme. Michaelis-Menten kinetic analysis of (b) OmpF1, (c) OmpF1Y, and (d) OmpF2. The metal-dependent net activities were obtained by the subtraction of the observed activities measured with free Zn ions from those in the presence of Zn-bound protein, in which the apo-protein exhibits no detectible activity. The data shown in (b), (c), and (d) represent the average and standard deviation of three independent experiments.

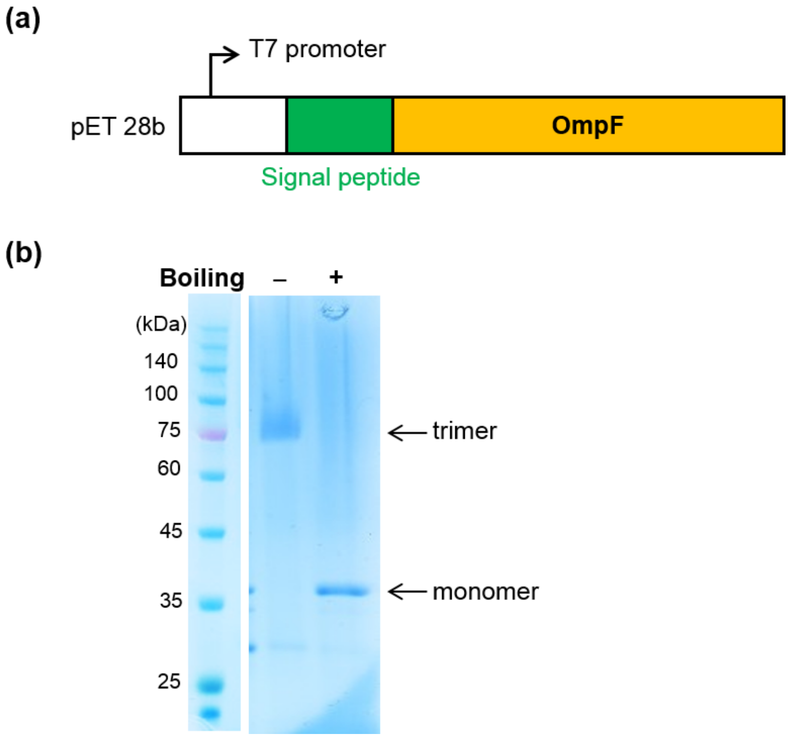

Supplementary Figure 7. Expression and translocation of the OmpF variants to the outer membrane. (a) The design of the OmpF expression vector by inserting a signal peptide at the N-terminus. (b) Representative SDS-PAGE of the OmpF variants extracted from *E. coli* cells (left) before and (right) after boiling. The data shown in (b) are representatives of at least ten trials, and all attempts to replicate were successful.

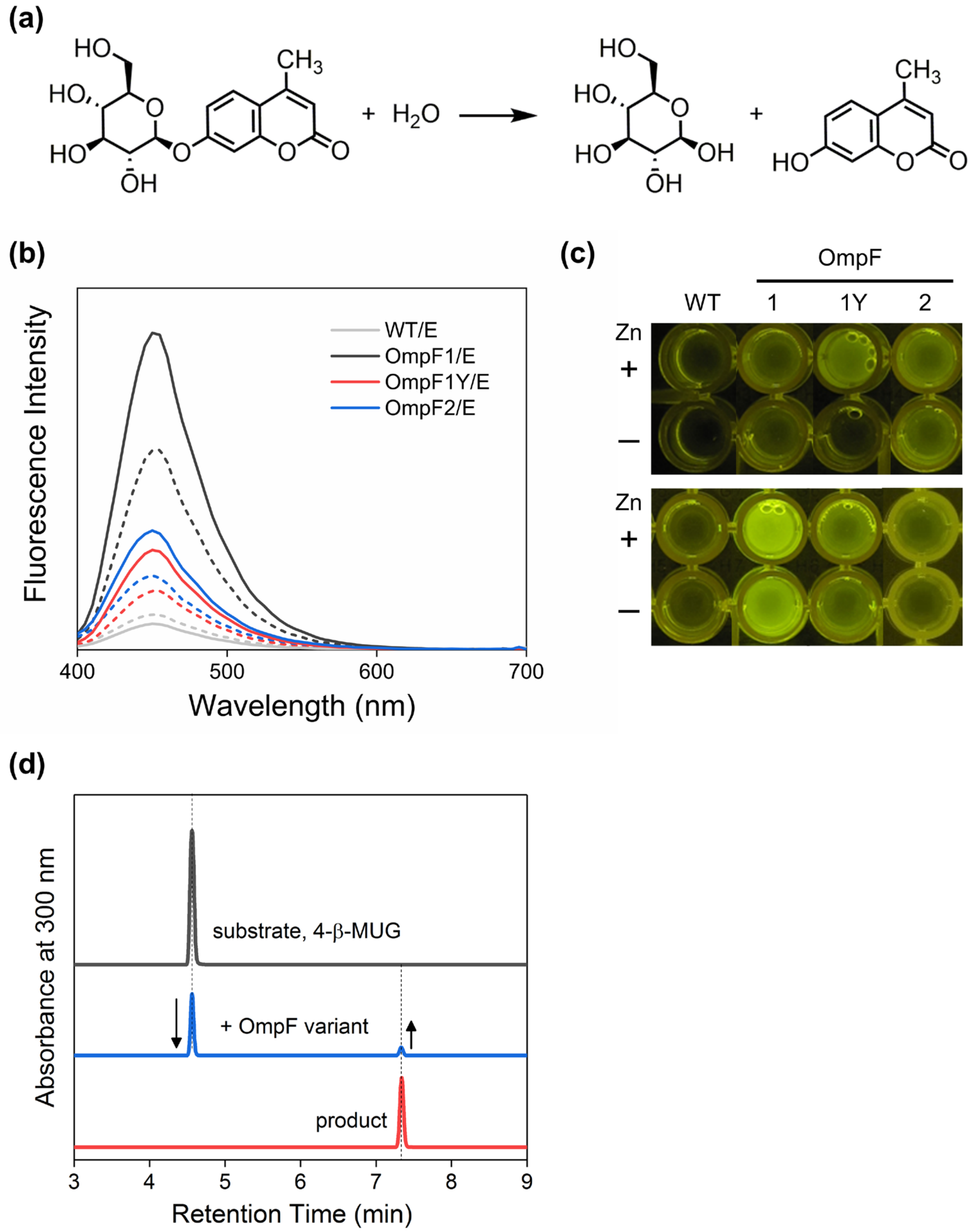

Supplementary Figure 8. Steady-state glycosidase activities of the OmpF variants with 4-β-MUG. (a) A reaction scheme. (b–c) The representative fluorescence spectra of the OmpF variants incubated with 4-β-MUG due to the formation of 4-methylumbelliferone as the hydrolyzed product. (b) The wild-type protein and OmpF variants after the D113E (/E) mutations. The solid and dotted lines indicate the proteins in the presence and absence of Zn ions, respectively. (c) Images of the samples described in Fig. 3c and S8b. (d) The representative HPLC trace of the reaction mixtures due to the consumption of 4-β-MUG and the formation of the hydrolyzed product, 4-methylumbelliferone.

(a)

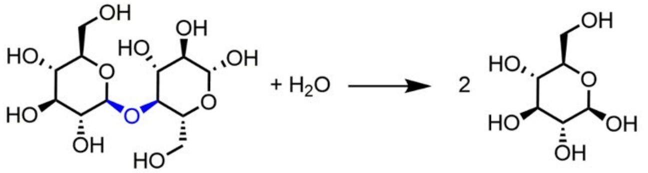

(b)

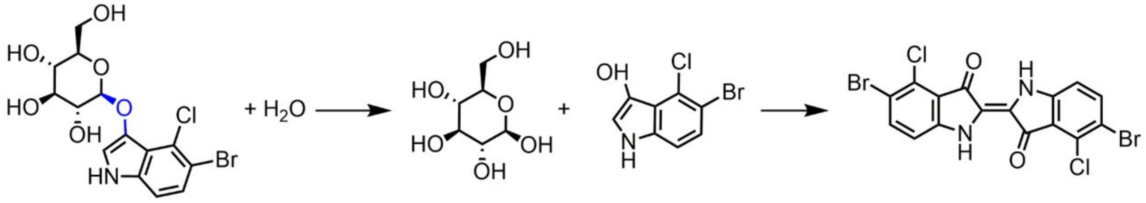

(c)

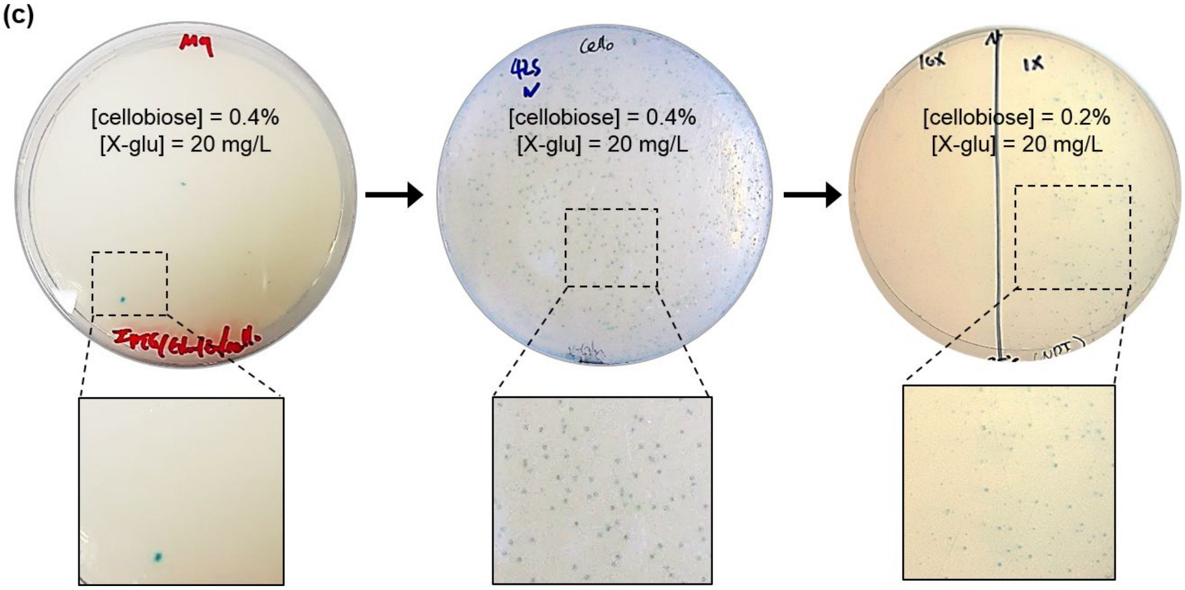

Supplementary Figure 9. Screening of whole-cell mutant libraries. The reaction schemes of the hydrolysis of (a) cellobiose and (b) X-Glu. (c) Representative agar plates to select artificial glycosidases with elevated glycosidase activity. Throughout the iterative rounds of screening, the concentration of cellobiose was adjusted gradually from 0.4% to 0.2%. The formation of blue colonies owing to the hydrolysis of two substrates were observed.

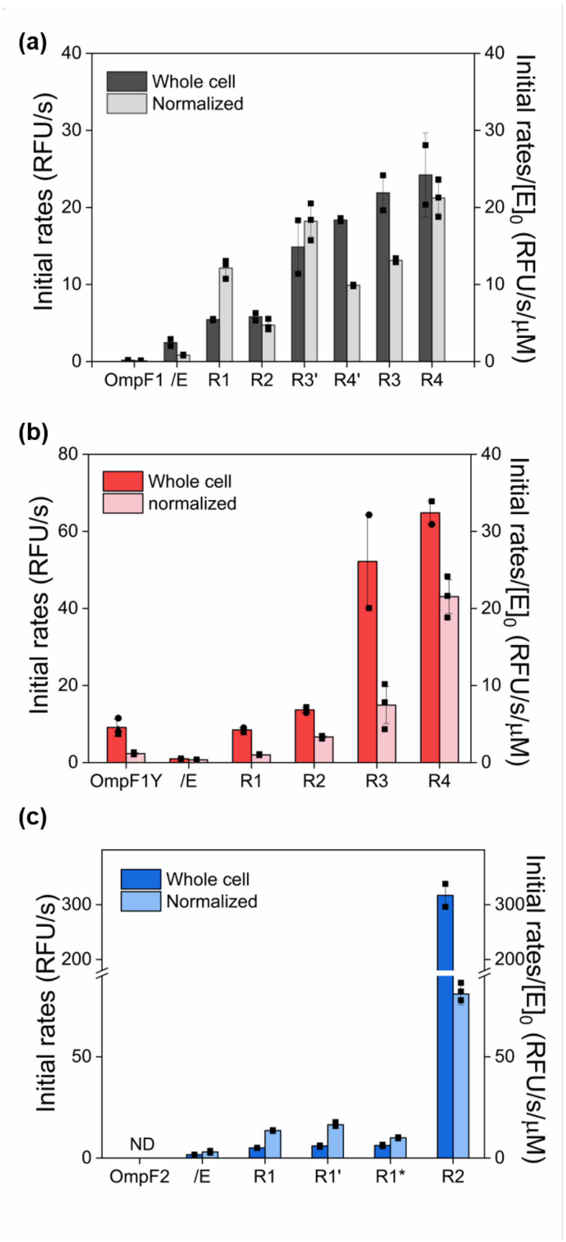

OmpF1	sequence
/E	D113E
R1	D113E/R42S
R2	D113E/R42S/Y106A
R3′	D113E/R42S/Y106A/A123E
R4′	D113E/R42S/Y106A/A123E/G120N
R3	D113E/R42S/Y106A/G120C
R4	D113E/R42S/Y106A/G120C/A123N

OmpF1Y	sequence
/E	D113E
R1	D113E/Y106R
R2	D113E/Y106R/R42E
R3	D113E/Y106R/R42E/G120S
R4	D113E/Y106R/R42E/G120S/A123V

OmpF2	sequence
/E	D113E
R1′	D113E/R82Y
R1	D113E/Y106H
R1*	D113E/R82Y/Y106H
R2	D113E/Y106H/R82C

Supplementary Figure 10. The screening results of (a) OmpF1, (b) OmpF1Y, and (c) OmpF2 as parent templates. (left) The whole-cell activity values were measured using 4-β-MUG, and the normalized activity values were obtained by dividing the whole-cell activity values by the protein concentration values of the cell lysates determined using SDS-PAGE. (right) The sequence of the screening results. R1–R4 indicate the rounds of selection. The prime and asterisk indicate the output from alternatively constructed mutant libraries and the addition of two screening hits, respectively. The fluorescence changes with OmpF2/E-R2 in (c) exceeded the detection limit, and we adjusted the gain for quantitative analysis. The whole cell data and normalized data shown in (a), (b), and (c) represent the average and standard deviation of at least two technical replicates of the experiments and three independent experiments, respectively. Source data are provided as a Source Data file.

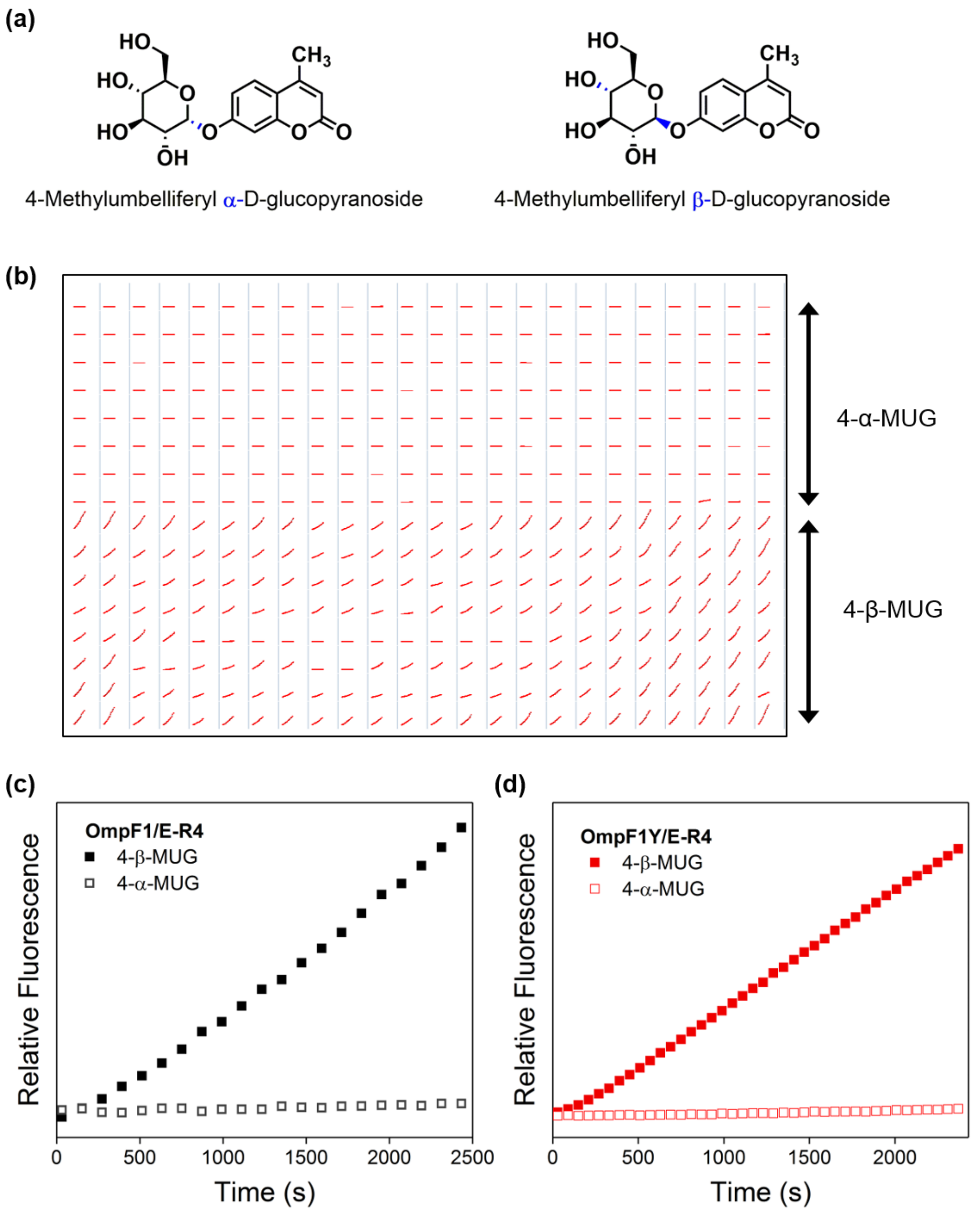

Supplementary Figure 11. Representative measurements of the stereoselective glycosidase activities of the OmpF variants. (a) Structures of 4-MUG having either a α- or β-glycosidic bond (4-α-MUG or 4-β-MUG, respectively). (b) Time-dependent fluorescence assays of 4-α-MUG or 4-β-MUG added to the duplicates of whole-cell libraries. The results of (c) OmpF1/E-R4 (d) OmpF1Y/E-R4.

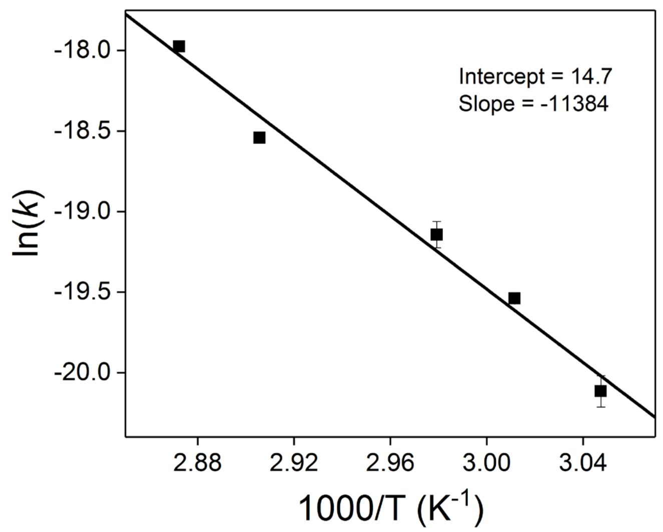

Supplementary Figure 12. Measurements of the uncatalyzed hydrolytic rates of 4-β-MUG. The data represent the average and standard deviation of three independent experiments.

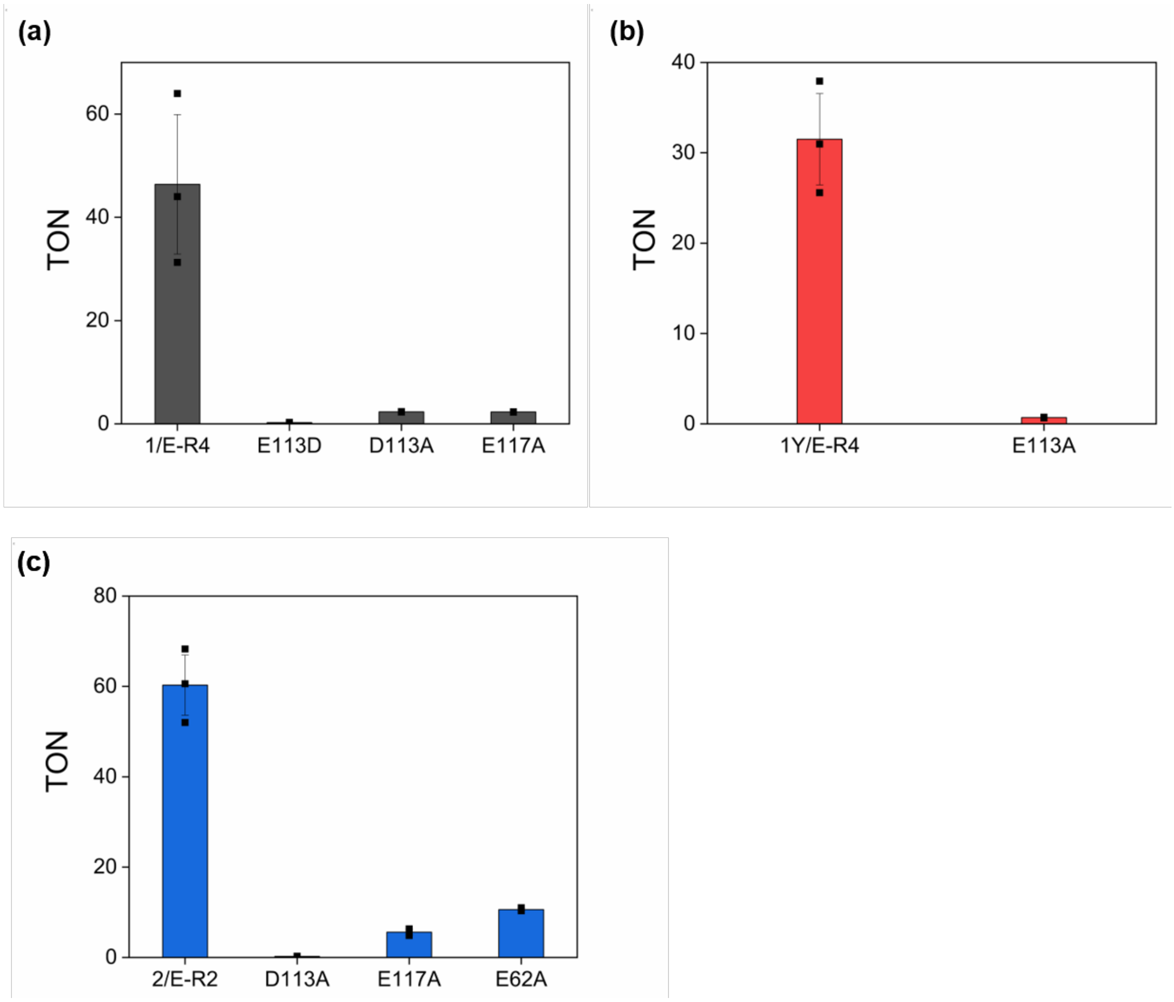

Supplementary Figure 13. The glycosidase activities of OmpF variants. One of acidic residues nearby the Zn-site in (a) OmpF1/E-R4 (b) OmpF1Y/E-R4, and (c) OmpF2/E-R2 variants are modified with alanine or aspartate. The data shown in (a), (b), and (c) represent the average and standard deviation of three independent experiments. Source data are provided as a Source Data file.

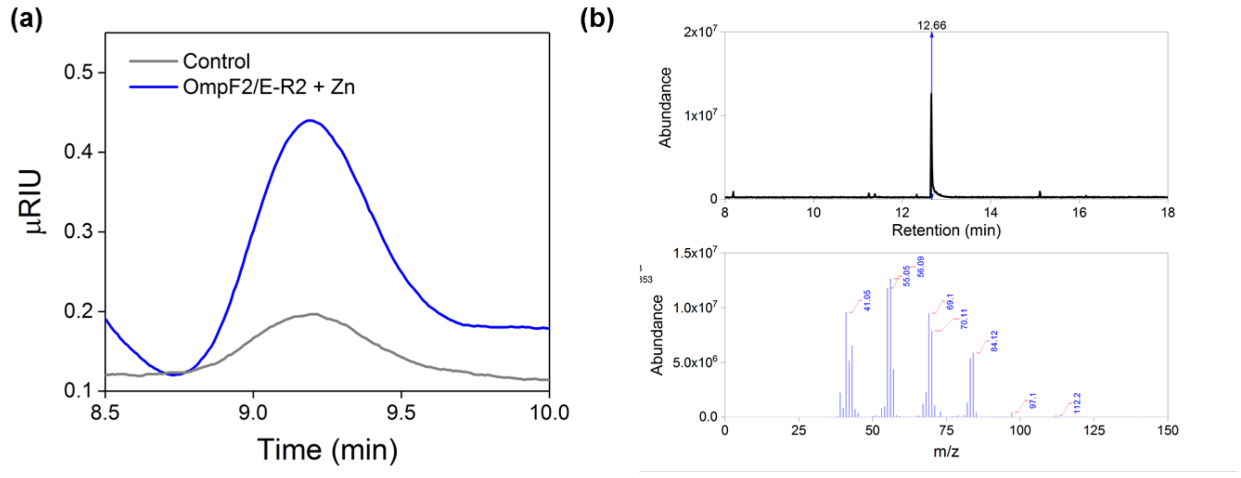

Supplementary Figure 14. The catalytic activities of OmpF variants with OG. Detection of (a) glucose by HPLC and (b) 1-octanol by GC-MS.

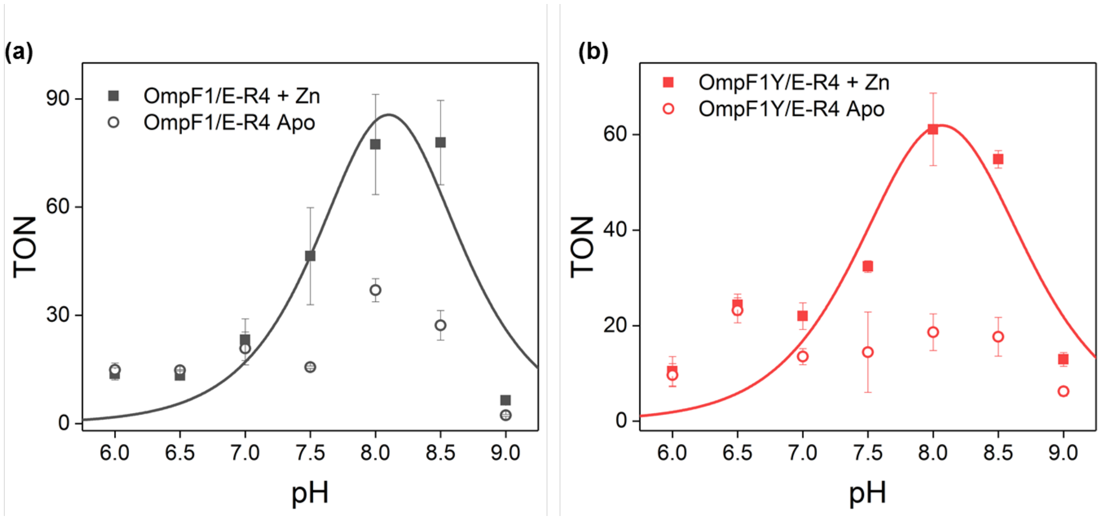

Supplementary Figure 15. The pH-dependent glycosidase activities of OmpF variants. (a) OmpF1/E-R4 (b) OmpF1Y/E-R4. The data represent the average and standard deviation of three independent experiments.

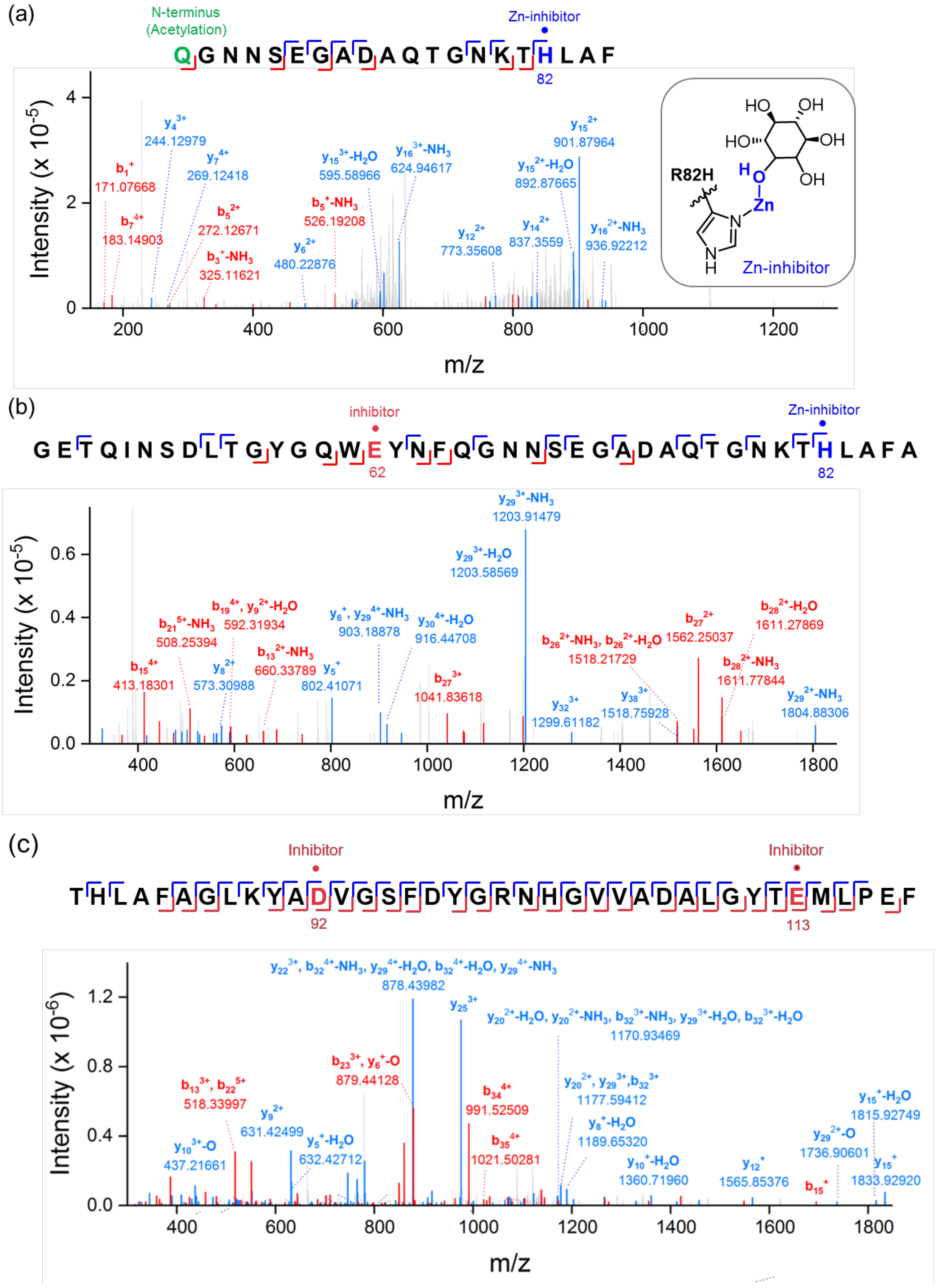
(a)
N-terminus (Acetylation)
Zn-inhibitor
Q G N N S E G A D A Q T G N K T H L A F
82
Intensity (x 10^{-5})
m/z
b_1^{+} 171.07668
b_7^{4+} 183.14903
y_4^{3+} 244.12979
y_7^{4+} 269.12418
b_5^{2+} 272.12671
b_3^{+}-NH_3 325.11621
y_6^{2+} 480.22876
b_5^{+}-NH_3 526.19208
y_{15}^{3+}-H_2O 595.58966
y_{16}^{3+}-NH_3 624.94617
y_{12}^{2+} 773.35608
y_{14}^{2+} 837.3559
y_{15}^{2+}-H_2O 892.87665
y_{15}^{2+} 901.87964
y_{16}^{2+}-NH_3 936.92212
R82H
Zn
Zn-inhibitor
HO
OH
(b)
inhibitor
Zn-inhibitor
G E T Q I N S D L T G Y G Q W E Y N F Q G N N S E G A D A Q T G N K T H L A F A
62
82
Intensity (x 10^{-5})
m/z
b_{15}^{4+} 413.18301
b_{21}^{5+}-NH_3 508.25394
b_{19}^{4+}, y_9^{2+}-H_2O 592.31934
y_8^{2+} 573.30988
b_{13}^{2+}-NH_3 660.33789
y_5^{+} 802.41071
y_6^{+}, y_{29}^{4+}-NH_3 903.18878
y_{30}^{4+}-H_2O 916.44708
b_{27}^{3+} 1041.83618
y_{29}^{3+}-NH_3 1203.91479
y_{29}^{3+}-H_2O 1203.58569
y_{32}^{3+} 1299.61182
b_{26}^{2+}-NH_3, b_{26}^{2+}-H_2O 1518.21729
y_{38}^{3+} 1518.75928
b_{27}^{2+} 1562.25037
b_{28}^{2+}-H_2O 1611.27869
b_{28}^{2+}-NH_3 1611.77844
y_{29}^{2+}-NH_3 1804.88306
(c)
Inhibitor
Inhibitor
T H L A F A G L K Y A D V G S F D Y G R N H G V V A D A L G Y T E M L P E F
92
113
Intensity (x 10^{-6})
m/z
y_{10}^{3+}-O 437.21661
b_{13}^{3+}, b_{22}^{5+} 518.33997
y_9^{2+} 631.42499
y_5^{+}-H_2O 632.42712
y_{22}^{3+}, b_{32}^{4+}-NH_3, y_{29}^{4+}-H_2O, b_{32}^{4+}-H_2O, y_{29}^{4+}-NH_3 878.43982
b_{23}^{3+}, y_6^{+}-O 879.44128
y_{25}^{3+}
b_{34}^{4+} 991.52509
b_{35}^{4+} 1021.50281
y_{20}^{2+}-H_2O, y_{20}^{2+}-NH_3, b_{32}^{3+}-NH_3, y_{29}^{3+}-H_2O, b_{32}^{3+}-H_2O 1170.93469
y_{20}^{2+}, y_{29}^{3+}, b_{32}^{3+} 1177.59412
y_8^{+}-H_2O 1189.65320
y_{10}^{+}-H_2O 1360.71960
y_{12}^{+} 1565.85376
b_{15}^{+}
y_{29}^{2+}-O 1736.90601
y_{15}^{+}-H_2O 1815.92749
y_{15}^{+} 1833.92920

(d)

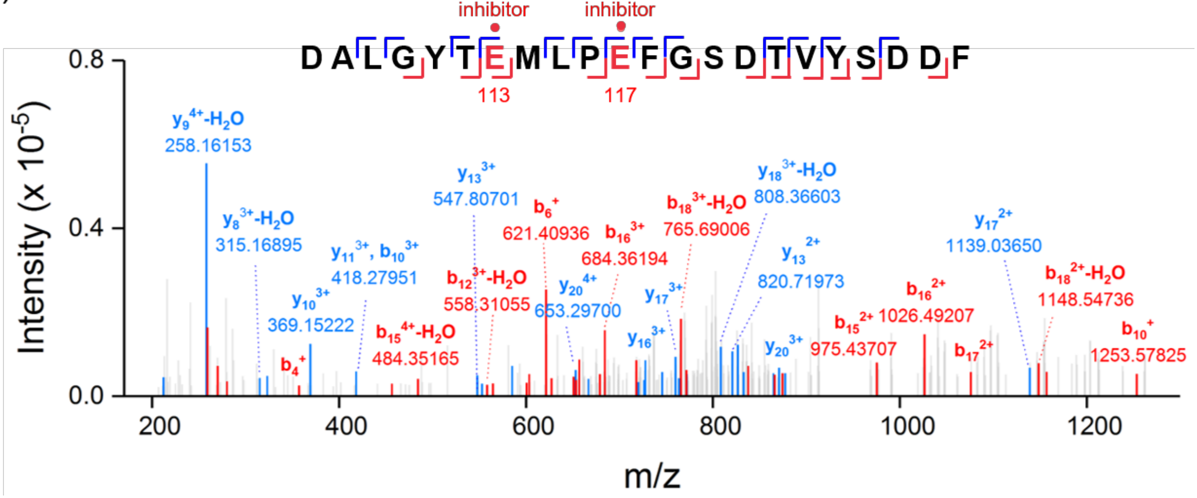

(e)

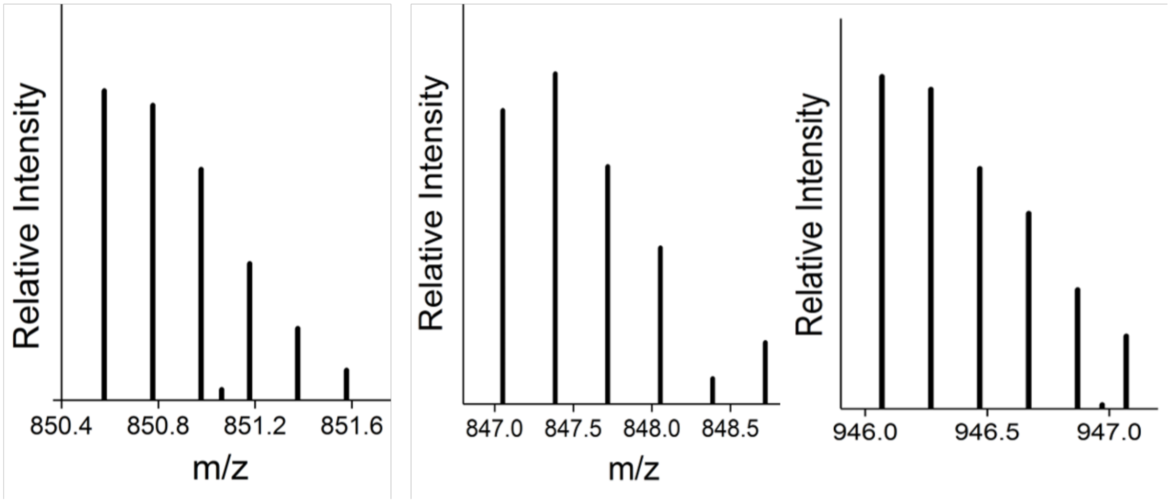

Supplementary Figure 16. Tandem LC/MS analysis of the trypsin-digested OmpF variants after incubation with CBE. (a) OmpF1/E-R4 and (b) OmpF1Y/E-R4 at Zn-complexed states. (c) OmpF1/E-R4 and (d) OmpF1Y/E-R4 at the apo-states. Only the representative fragments containing CBE inhibitor at the constriction zone are shown for clarity. (e) The fragments possessing Zn-moiety shows the characteristic patterns due to naturally occurring zinc isotopes (left: OmpF2/E-R2, middle: OmpF1/E-R4 right: OmpF1Y/E-R4).

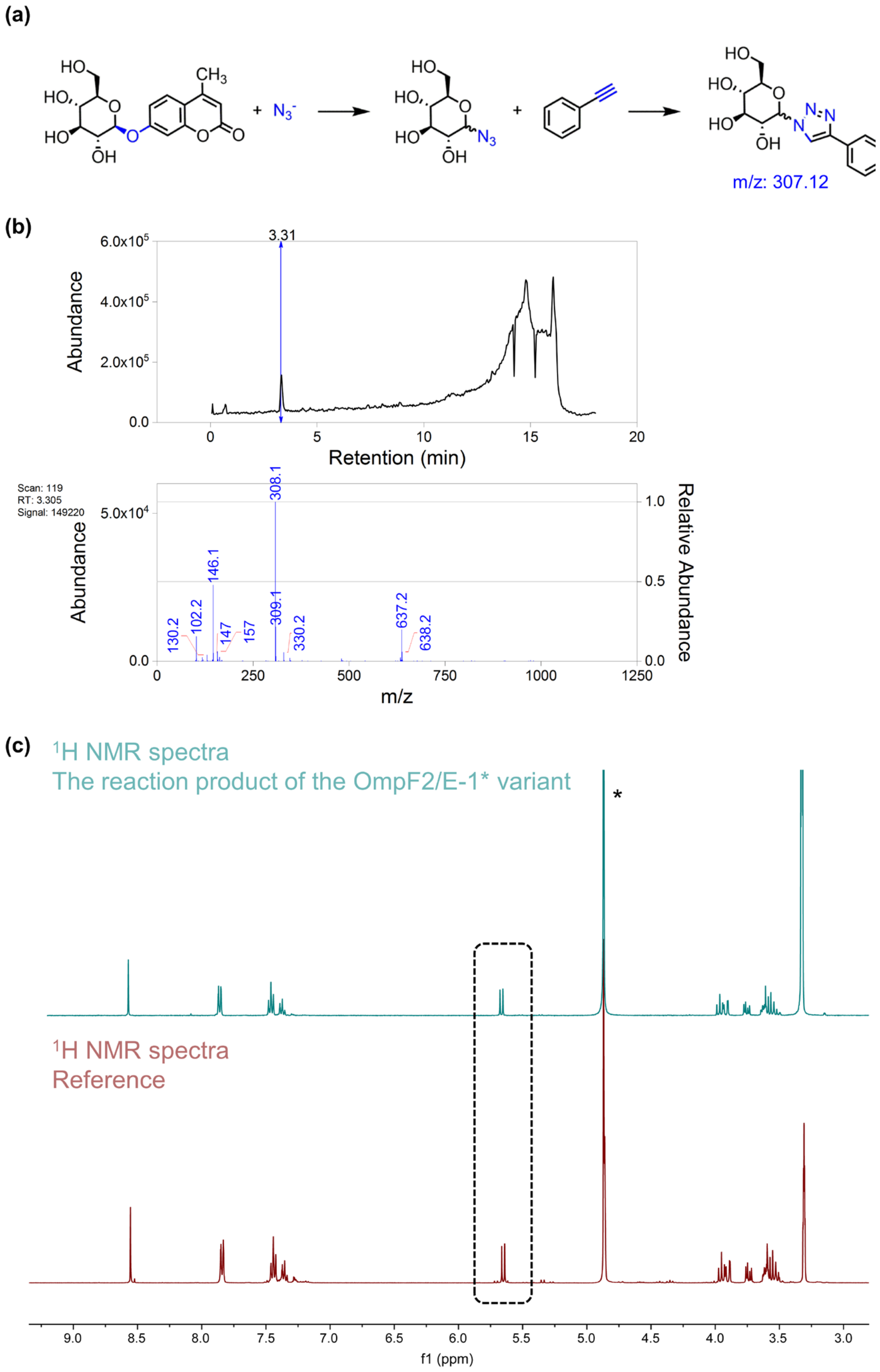

Supplementary Figure 17. Characterization of the glycosidase activity. (a) A reaction scheme. (b) ES-API mass spectrum. Calculated *m/z* for $C_{14}H_{17}N_3O_5^+$ $[M+H]^+$: 308.12; observed: 308.1. (c) ^1H NMR spectra. (top) The reaction product of the OmpF2/E-1* variant (bottom) 1-β-D-glucopyranosyl-4-phenyl-1H-1,2,3-triazole synthesized from 1-azido-1-deoxy-glucose as a reference. The dotted box highlights the chemical shift for a proton at the C1 anomeric carbon (5.65 ppm), indicative of a β-isomer. The asterisk in (c) indicates the residual H_2O solvent.

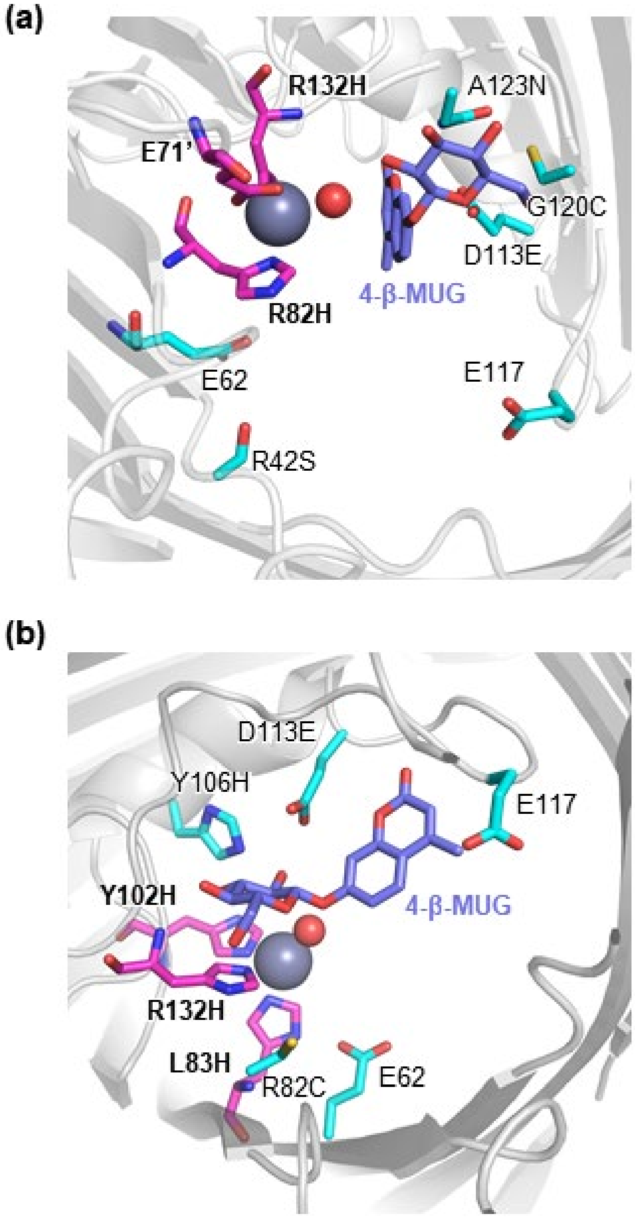

Supplementary Figure 18. Docking simulation of OmpF variants with 4-β-MUG. A possible mode of substrate-binding in (a) OmpF1/E-R4 and (b) OmpF2/E-R2. Zn-binding residues are labeled in bold. The selected positions for iterative sequence optimization or adjacently located acidic residues are shown with cyan sticks. The Zn atoms and Zn-bound water molecules are shown with light navy and red spheres, respectively. The bound 4-β-MUG substrate is shown with navy sticks.

Supplementary Table 1. Geometric analysis of the representative natural Zn-binding metalloproteins.

Proteins (PDB code)	Ligands	Zn−ligating atom (Å)	The $C_{\alpha}\cdots C_{\alpha}$ distance of ligating residues (Å)
Carbohydrate esterase (2CC0)	2His/Asp	2.1−2.8	7.1–8.5
Metallo-β-lactamase (2BC2)	3His	2.1−2.3	5.9–10.4
Thermolysin (4TLN)	2His/Glu	2.0−2.3	6.6–8.1
Carbonic anhydrase (3K34)	3His	2.0	5.5−8.1
Thermolysin (4N4E)	2His/Glu	2.0	6.6−8.4
Carboxypeptidase A (1M4L)	2His/Glu	2.0−2.3	6.0−7.2
Leucine aminopeptidase (1LAM)	Asp/Glu/Lys	2.0−2.2	7.6−10.5
	2Asp/Glu	2.0−2.1	5.2−8.7
Alkaline phosphatase (1ALK)	His/2Asp/Ser	1.8−2.2	3.8−7.8
	2His/Asp	2.0−2.3	6.1−8.7
Horse liver alcohol dehydrogenase (1HLD)	His/2Cys	2.2−2.3	7.4−8.1
	4Cys	2.3−2.4	5.1−7.4
Zinc finger protein (4M9V)	2His/2Cys	2.0−2.3	5.7−10.3
	2His/2Cys	2.0−2.3	5.7−9.8
Glyoxalase (2XF4)	2His/2Asp	2.1−2.4	3.8–10.6

Supplementary Table 2. Custom-designed DNA primers for (a) site-directed mutagenesis and (b) saturation mutagenesis.

(a)

Variants	Mutations	Primers
R82H/Y102H/R132H (OmpF1)	R82H	5′-GGTAACAAA ACGCATCTGGCATTCGCG G-3′<br>5′-CCGCGAATGCCAGATGCGTTTTGTTACC-3′
	Y102H	5'-GATTACGGCCGTAACCACGGTGTGGTTTATG-3'<br>5'-CATAAACC CACCGTGGTTACGGCCGTAATC-3'
	R132H	5′-CTTCGTTGGTCATCATGGCGGCGTTGCTAC-3′<br>5′-GTAGCAACGCCGCCATGATGACCAACGAAG-3′
L83H/Y102H/R132H (OmpF2)	L83H	5′-GTAACAAAACGCGTCATGCATTCGCGGGTC-3′<br>5′-GACCCGCGAATGCATGACGCGTTTTGTTAC-3′
	Y102H	5'-GATTACGGCCGTAACCACGGTGTGGTTTATG-3'<br>5'-CATAAACCACACCGTGGTTACGGCCGTAATC-3'
	R132H	5'-CTTCTTCGTTGGTCATGTTGGCGGCGTTG-3′<br>5′-CAACGCCGCCAACATGACCAACGAAGAAG-3'
R82H/R132H (OmpF1Y)	R82H	5′-GGTAACAAAACGCATCTGGCATTCGCGG-3′<br>5′-CCGCGAATGCCAGATGCGTTTTGTTACC-3′
	R132H	5′-CTTCTTCGTTGGTCATGTTGGCGGCGTTG-3′<br>5′-CAACGCCGCCAACATGACCAACGAAGAAG-3′
OmpF1/E, OmpF1Y/E, OmpF2/E	D113E	5′-GGGTTACACCGAGATGCTGCCAGAATTTGG-3′<br>5′-CCAAATTCTGGCAGCATCTCGGTGTAACCC-3′
OmpF1H/E/R42S/Y106A	R42S	5′-GACATGACCTATGCCAGCCTTGGTTTTAAAG-3′<br>5′-CTTTAAAACCAAGGCTGGCATAGGTCATGTC-3′
	Y106A	5′-GGTGTGGTTGCGGATGCACTGGGTTAC-3′<br>5′-GTAACCCAGTGCATCCGCAACCACACC-3′
OmpF1Y/E/Y106R/R42E	Y106R	5′-GGTGTGGTTCGTGATGCACTGGGTTAC-3′<br>5′-GTAACCCAGTGCATCACGAACCACACC-3′
	R42E	5′-GACATGACCTATGCCGAACTTGGTTTTAAAG-3′<br>5′-CTTTAAAACCAAGTTCGGCATAGGTCATGTC-3′
OmpF1Y/E/R42H/Y106A	R42H	5′-GACATGACCTATGCCCATCTTGGTTTTAAAG-3′<br>5′-CTTTAAAACCAAGATGGGCATAGGTCATGTC-3′
	Y106A	5′-GGTGTGGTTGCGGATGCACTGGGTTAC-3′<br>5′-GTAACCCAGTGCATCCGCAACCACACC-3′
OmpF2	R82Y	5′-GTAACAAAACGTACCATGCATTCGCG-3′<br>5′-CGCGAATGCATGGTACGTTTTGTTAC-3′
	Y106H	5′-GGTGTGGTTCACGATGCACTGGG-3′<br>5′-CCCAGTGCATCGTGAACCACACC-3′
His-tag deletion (5’-phospholyration)		5′-CATATGGAAATCTATAACAAAGATGGCAAC-3′<br>5′-TATATCTCCTTAAAGTTAAACAAAATTATTC-3′

(b)

Variants	Mutations	Primers
Y106X	NDT	5′-GGTGTGGTTNDTGATGCACTGGG-3′<br>5′-CCCAGTGCATCAHNAACCACACC-3′
	VHG	5′-GGTGTGGTTVHGGATGCACTGG-3′<br>5′-CCAGTGCATCCDBAACCACACC-3′
R42S	NDT	5′-GACATGACCTATGCCNDTCTTGGTTTTAAAG-3′<br>5′-CTTTAA AACCAAGAHNGGCATAGGTCATGTC-3′
	VHG	5′-GACATGACCTATGCCVHGCTTGGTTTTAAAG-3′<br>5′-CTTTAA AACCAAGCDBGGCATAGGTCATGTC-3′
R82X	NDT	5′-GGTAACAAAACGNDTCATGCATTCGCG-3′<br>5′-CGCGAATGCATGAHNCGTTTTGTTACC-3′
	VHG	5′-GGTAACAAAACGVHGCATGCA TTC GCG-3′<br>5′-CGCGAATGCATGCDBCGTTTT GTT ACC-3′
Y102X	NDT	5′-GATTACGGCCGTAACNDTGGTGTGG-3′<br>5′-CCACACCAHNGTTACGGCCGTAATC-3′
	VHG	5′-GATTACGGCCGTAACVHGGGTGTGG-3′<br>5′-CCACACCCDBGTTACGGCCGTAATC-3′
S125X	NDT	5′-GGTGGTGATACTGCATACNDTGATGACTTC-3′<br>5′-GAAGTCATCAHNGTATGCAGTATCACCACC-3′
	VHG	5′-GGTGGTGATACTGCATACVHGGATGACTTC-3′<br>5′-GAAGTCATCCDBGTATGCAGTATCACCACC-3′
G120X	NDT	5′-GAGATGCTGCCAGAATTTGGTNDTGATACT-3′<br>5′-AGTATCAHNACCAAATTCTGGCAGCATCTC-3′
	VHG	5′-GAGATGCTGCCAGAATTTGGTVHGGATACT-3′<br>5′-AGTATCCDBACCAAATTCTGGCAGCATCTC-3′
A123X	NDT	5′-GATACTNDTTACAGCGATGACTTCTTC-3′<br>5′-GAAGAAGTCATCGCTGTAAHNAGTATC-3′
	VHG	5′-GATACTVHGTACAGCGATGACTTCTTC-3′<br>5′-GAAGAAGTCATCGCTGTACDBAGTATC-3′
D113X	NDT	5′-GTTACACCNDTATGCTGCCAGAATTTGG-3′<br>5′-CCAAATTCTGGCAGCATAHNGGTGTAAC-3′
	VHG	5′-GTTACACCVHGATGCTGCCAGAATTTGG-3′<br>5′-CCAAATTCTGGCAGCATCDBGGTGTAAC-3′

Supplementary Table 3. Crystallographic data and refinement statistics

	OmpF1 (7FDY)	OmpF2 (7FF7)	OmpF2 (Zn SAD)
**Data collection**			
Wavelength (Å)	0.979	0.979	1.282
Space group	C2	C2	C2
Unit cell length (a, b, c, Å)	128.6, 140.4, 110.9	153.7, 112.0, 110.8	154.1, 112.4, 111.2
Unit cell angle (α, β, γ, °)	90.0, 120.0, 90.0	90.0, 110.9, 90.0	90.0, 110.9, 90.0
Resolution (Å)	30–3.10 (3.27–3.10)	50–3.38 (3.44–3.38)	50–3.98 (4.05–3.98)
R_{merge} (%)	10.9 (77.7)	12.5 (45.8)	19.8 (85.1)
$I / \sigma I$	10.33 (1.6)	32.5 (8.0)	23.6 (4.0)
Completeness (%)	98.3 (98.1)	99.7 (100.0)	99.6 (100.0)
Redundancy	3.7 (3.7)	7.2 (7.5)	3.8 (3.7)
$CC_{1/2}$ (%)	99.7 (83.9)	98 (98)	98 (82)
**Refinement**			
Resolution (Å)	29.13-3.1 (3.2–3.1)	38.05–3.38 (3.51–3.38)	
No. reflections	30643 (1532)	23204 (1206)	
R_{work} / R_{free}[b]	0.270/0.296	0.290/0.326	
No. atoms			
Protein	7599	7038	
Ligand/ion	3	3	
Water	3	14	
B-factors			
Protein	86.5	101.43	
Ligand/ion	82.2	80.99	
Water	58.7	62.44	
R.m.s. deviations			
Bond lengths (Å)	0.01	0.005	
Bond angles (°)	1.01	1.37	
Ramachandran plot (%)			
Favored	94.21	87.8	
Allowed	5.79	10.1	
Outliers	0.0	2.1	

A single crystal was used for each data collection. Values in parentheses are for the highest-resolution shell.

Supplementary Table 4. The geometric parameters of the Zn-binding sites in OmpF1 (PDB 7FDY) and OmpF2 (PDB 7FF7).

The distance of key residues

Interatomic distance (Å)	OmpF1	OmpF2
Zn−ligating atoms	2.0–2.4	2.1–2.3
C_α–C_α of ligating atoms	6.6–9.2	5.4–8.9
Zn-bound H_2O and D113	7.9–9.5	4.7–5.1
Zn-bound H_2O and E117	10.8–13.3	10.0–11.7
Zn-bound H_2O and E62	8.2–10.2	6.2–7.3
Oxygen atoms between D113 and E117	6.4–12.0	6.3–11.3
Oxygen atoms between D113 and E62	11.0–13.5	11.2–132
Oxygen atoms between D113 and E71	11.6–13.5	10.9–13.2
Oxygen atoms between E117 and E71	12.8–16.1	11.9–15.1
Oxygen atoms between E117 and E62	12.6–14.9	11.8–14.7
Oxygen atoms between E71and E62	8.0–10.4	9.4–10.6

The angles of key residues

	OmpF1	OmpF2
The angles of O/N−Zn−N of Zn-ligating residues (°)	94.9–119.4	71.3–99.4

Supplementary Table 5. Steady-state kinetic analysis of the OmpF variants. The rates for either apo or Zn-bound samples were measured in triplicate.

Esterase activity with *p*-NPA

	k_{cat} (s^{-1})	K_M (mM)	k_{cat}/K_M (s^{-1} M^{-1})
OmpF1	0.006 ± 0.001	0.5 ± 0.4	12 ± 8
OmpF1Y	0.026 ± 0.004	2.5 ± 0.8	10 ± 2
OmpF2	0.030 ± 0.004	3 ± 1	12 ± 3

β-lactamase activity with nitrocefin

	k_{cat} (s^{-1})	K_M (mM)	k_{cat}/K_M (s^{-1} M^{-1})
OmpF1	0.017 ± 0.004	1.4 ± 0.6	12 ± 2
OmpF1Y	0.02 ± 0.01	2 ± 1	15 ± 3
OmpF2	0.025 ± 0.005	1.7 ± 0.6	14 ± 2

Supplementary Table 6. Glycosidase activities of the OmpF variants with 4-β-MUG. The steady-state kinetic parameters were obtained as second-order rate constants (k_2) with the OmpF variants (10 μM). The uncatalyzed rate constant (k_{uncat}) was measured to be 3.72(± 0.63) × 10^{-9} min^{-1} as shown in Supplementary Fig. 12. The rates were measured in triplicate.

	Zn	k_2 (min^{-1} M^{-1})	k_2/k_{uncat} (M^{-1})
OmpF1/E-R4	–	3.7 ± 0.1	0.99(± 0.1) x 10^9
	+	6.8 ± 0.3	1.83(± 0.3) x 10^9
OmpF1Y/E-R4	–	2.8 ± 0.7	0.76(± 0.1) x 10^9
	+	5.6 ± 0.3	1.51(± 0.2) x 10^9
OmpF2/E-R2	–	4.4 ± 0.1	1.2(± 0.2) x 10^9
	+	10.4 ± 0.3	2.8(± 0.3) x 10^9

Supplementary Table 7. ICP-MS results of OmpF2/E-R2. The molar ratio of metal to protein were obtained from two independently prepared batches of the samples.

Molar ratio	Cu	Zn
WT	N.D./1.38	0.560/0.753
OmpF2/E-R2	N.D./1.40	2.105/2.118

N.D.; not detected.

Supplementary Table 8. A list of fragments conjugated with CBE in tandem LC/MS analysis. The conjugated residues were grouped by the proximity to the Zn site and the relevance to the active site.

Zn-bound states

Protein	Proximal to Zn site	Non-constriction zone
WT	ND	D37, D92, E212, D221
D113E	ND	D221
OmpF1/E-R4	E62, D113E, E117, ZnOH:R82H	E29, D37, E48, D97, D107, D121, D149, D172, E181, E183, D195, E284, D290, E296, D312, D319
OmpF1Y/E-R4	E62, ZnOH:R82H	E29, D37, E42, D221
OmpF2/E-R2	E62, E71, ZnOH:Y102H	E29, D37, E48, D54, D74, D107, D121, D172, E181, E183, D195, E233, D266, E284, D288, E296, D312, D328

ND, Not detected

Apo-states

Protein	Proximal to Zn site	Non-constriction zone
WT	ND	D312
D113E	ND	ND
OmpF1/E-R4	E71, D113E	D92, D172, E181, E183, D195, E201, D221, D282, E284, D312
OmpF1Y/E-R4	D113E, E117	D92, E201, E212, D221, D290
OmpF2/E-R2	D113E, E117	E29, D37, D121, D172, E181, E212, D221, E233

ND, Not detected
