# Supplementary material for: Design and directed evolution of noncanonical β-stereoselective metalloglycosidases
Source: Nat Commun. 2022 Nov 11;13:6844. doi: 10.1038/s41467-022-34713-8 (PMC9652281; doi:10.1038/s41467-022-34713-8)
Supplement: Supplementary file 3 — Reporting Summary [file 41467_2022_34713_MOESM3_ESM.pdf]

nature portfolio

Corresponding author(s): Woon Ju Song

Last updated by author(s): Nov 4, 2022

Reporting Summary

Nature Portfolio wishes to improve the reproducibility of the work that we publish. This form provides structure for consistency and transparency in reporting. For further information on Nature Portfolio policies, see our Editorial Policies and the Editorial Policy Checklist.

Statistics

For all statistical analyses, confirm that the following items are present in the figure legend, table legend, main text, or Methods section.

n/a	Confirmed	
☐	☒	The exact sample size (*n*) for each experimental group/condition, given as a discrete number and unit of measurement
☐	☒	A statement on whether measurements were taken from distinct samples or whether the same sample was measured repeatedly
☒	☐	The statistical test(s) used AND whether they are one- or two-sided<br>*Only common tests should be described solely by name; describe more complex techniques in the Methods section.*
☒	☐	A description of all covariates tested
☒	☐	A description of any assumptions or corrections, such as tests of normality and adjustment for multiple comparisons
☐	☒	A full description of the statistical parameters including central tendency (e.g. means) or other basic estimates (e.g. regression coefficient) AND variation (e.g. standard deviation) or associated estimates of uncertainty (e.g. confidence intervals)
☒	☐	For null hypothesis testing, the test statistic (e.g. *F*, *t*, *r*) with confidence intervals, effect sizes, degrees of freedom and *P* value noted<br>*Give P values as exact values whenever suitable.*
☒	☐	For Bayesian analysis, information on the choice of priors and Markov chain Monte Carlo settings
☒	☐	For hierarchical and complex designs, identification of the appropriate level for tests and full reporting of outcomes
☒	☐	Estimates of effect sizes (e.g. Cohen's *d*, Pearson's *r*), indicating how they were calculated

Our web collection on statistics for biologists contains articles on many of the points above.

Software and code

Policy information about availability of computer code

Data collection	Kinetic data were collected using BioTek Gen5 software (version 3.0.2). Liquid chromatography (LC) and LC-MS data were obtained using Agilent Chemstation software version C.01.09 and B.04.03, respectively. Tandem LC/MS data were collected using Xcaliber software (version 4.3). NMR data were collected using VnmrJ software (version 4.2). GC-MS data were collected using Xcalibur software (version 4.0.0.29). Computational docking was performed using AutoDock Vina software (version 1.1.2.).
Data analysis	X-ray crystallography: HKL2000_v721 and XDS (data integration), CCP4i 7.0.078, Phenix 1.14-3260 (scaling/model building/refinement), Coot v.0.8.9.2 (refinement) and PyMOL version 2.2.3 (molecular graphics); LC-MS/MS: Proteome Discoverer 2.3; Kinetic analysis: Origin 2021 (64-bit) 9.8.0.200 (academic); NMR data: MestReNova x64-14.2.0.

For manuscripts utilizing custom algorithms or software that are central to the research but not yet described in published literature, software must be made available to editors and reviewers. We strongly encourage code deposition in a community repository (e.g. GitHub). See the Nature Portfolio guidelines for submitting code & software for further information.

Data

Policy information about availability of data

All manuscripts must include a data availability statement. This statement should provide the following information, where applicable:

- Accession codes, unique identifiers, or web links for publicly available datasets
- A description of any restrictions on data availability
- For clinical datasets or third party data, please ensure that the statement adheres to our policy

All the data generated in this study are available within the main text, the Supplementary Information file; source data are provided in the Source Data file. Data is also available from the corresponding author upon request. Coordinates and structure factors for the reported crystal structures in this work were deposited in the RCSB PDB under accession numbers 7FDY (OmpF1) and 7FF7 (OmpF2).

Human research participants

Policy information about studies involving human research participants and Sex and Gender in Research.

Reporting on sex and gender	N/A
Population characteristics	N/A
Recruitment	N/A
Ethics oversight	N/A

Note that full information on the approval of the study protocol must also be provided in the manuscript.

Field-specific reporting

Please select the one below that is the best fit for your research. If you are not sure, read the appropriate sections before making your selection.

- [x] Life sciences
- [ ] Behavioural & social sciences
- [ ] Ecological, evolutionary & environmental sciences

For a reference copy of the document with all sections, see nature.com/documents/nr-reporting-summary-flat.pdf

Life sciences study design

All studies must disclose on these points even when the disclosure is negative.

Sample size	No statistical methods were used to predetermine the sample size. The kinetic measurements under in vitro conditions were conducted in triplicate throughout this study.
Data exclusions	No data were excluded.
Replication	All experimental trials to replicate were successful, and the data shown in the manuscript are the representative of experiments repeated at least once. At least two independent replicates were done for screening. All experiments were conducted in triplicate for kinetic analysis of enzymes under in vitro conditions.
Randomization	Qualitative analysis of artificially designed enzymes herein is irrelevant to randomization.
Blinding	Blinding was not relevant to this study since we made no statistical comparison.

Reporting for specific materials, systems and methods

We require information from authors about some types of materials, experimental systems and methods used in many studies. Here, indicate whether each material, system or method listed is relevant to your study. If you are not sure if a list item applies to your research, read the appropriate section before selecting a response.

Materials & experimental systems

n/a	Involved in the study
☒	☐ Antibodies
☒	☐ Eukaryotic cell lines
☒	☐ Palaeontology and archaeology
☒	☐ Animals and other organisms
☒	☐ Clinical data
☒	☐ Dual use research of concern

Methods

n/a	Involved in the study
☒	☐ ChIP-seq
☒	☐ Flow cytometry
☒	☐ MRI-based neuroimaging
